# Retroperitoneal free air after peroral endoscopic myotomy: It can happen!

**DOI:** 10.1016/j.igie.2025.06.007

**Published:** 2025-07-18

**Authors:** Malique Delbrune, Thomas Enke, Mohammad Bilal

**Affiliations:** 1 Department of Internal Medicine, University of Colorado Anschutz Medical Campus, Aurora, Colorado, USA; 2 Division of Gastroenterology and Hepatology, University of Colorado Anschutz Medical Campus, Aurora, Colorado, USA

A 60-year-old male with type III achalasia previously treated with botulinum toxin injections underwent a peroral endoscopic myotomy (POEM). A selective circular myotomy was performed for the spastic segment, and a full-thickness myotomy was performed at the lower esophageal sphincter. The final myotomy measured 10 cm and extended 1 cm into the gastric cardia. The mucosal incision was closed with endoclips (Fig. 1). The procedure was challenging owing to severe submucosal fibrosis; however, no intraprocedural adverse events (AEs) occurred. He received intravenous antibiotics following the procedure, consistent with standard practices at our institution. The postprocedure course was complicated by severe chest and abdominal pain and tachypnea. He was afebrile, and the abdominal examination result was benign. The chest radiograph revealed lucency in the inferior mediastinum, consistent with pneumomediastinum (Fig. 2), as expected post-POEM. Given symptom severity, a computed tomography scan with intravenous contrast of the chest and abdomen was obtained, which revealed small-volume pneumomediastinum and retroperitoneal free air (Figs. 3 and 4). Although pneumomediastinum and pneumoperitoneum are expected findings post-POEM, retroperitoneal free air is less common.

**Figure 1:**
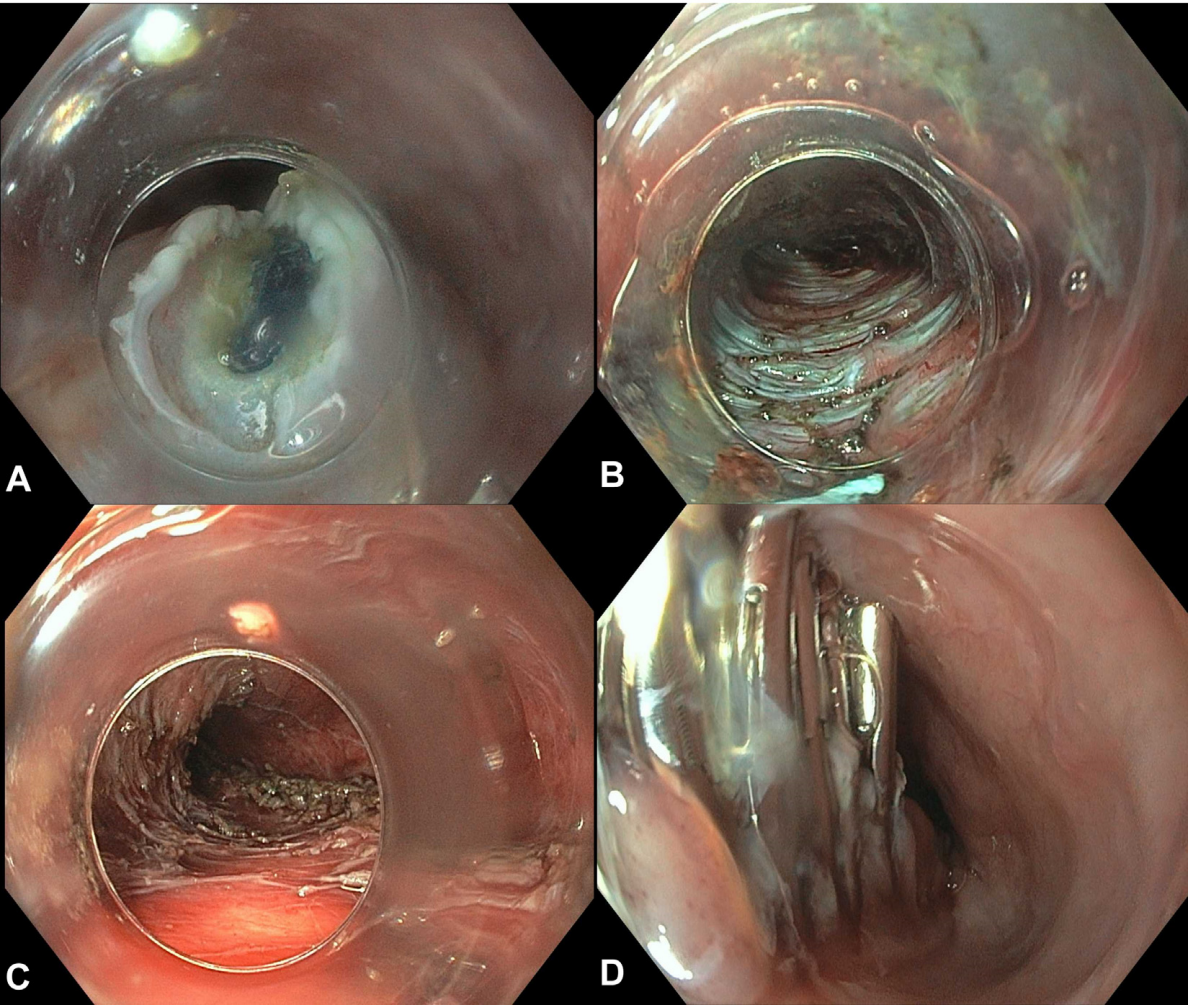
Endoscopic images of the key steps during the peroral endoscopic myotomy including mucosal incision (**A**), submucosal tunnel (**B**), full-thickness myotomy (**C**), and mucosal incision closure (**D**).

**Figure 2:**
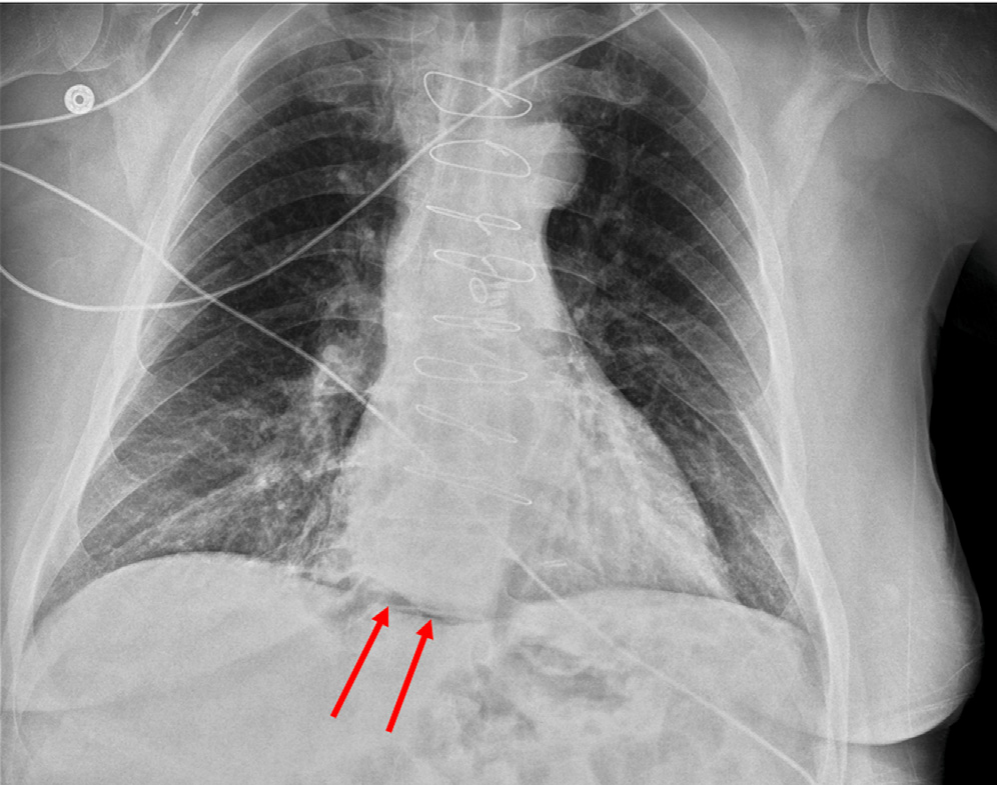
Chest radiograph with lucency in inferior mediastinum (*red arrows*).

**Figure 3:**
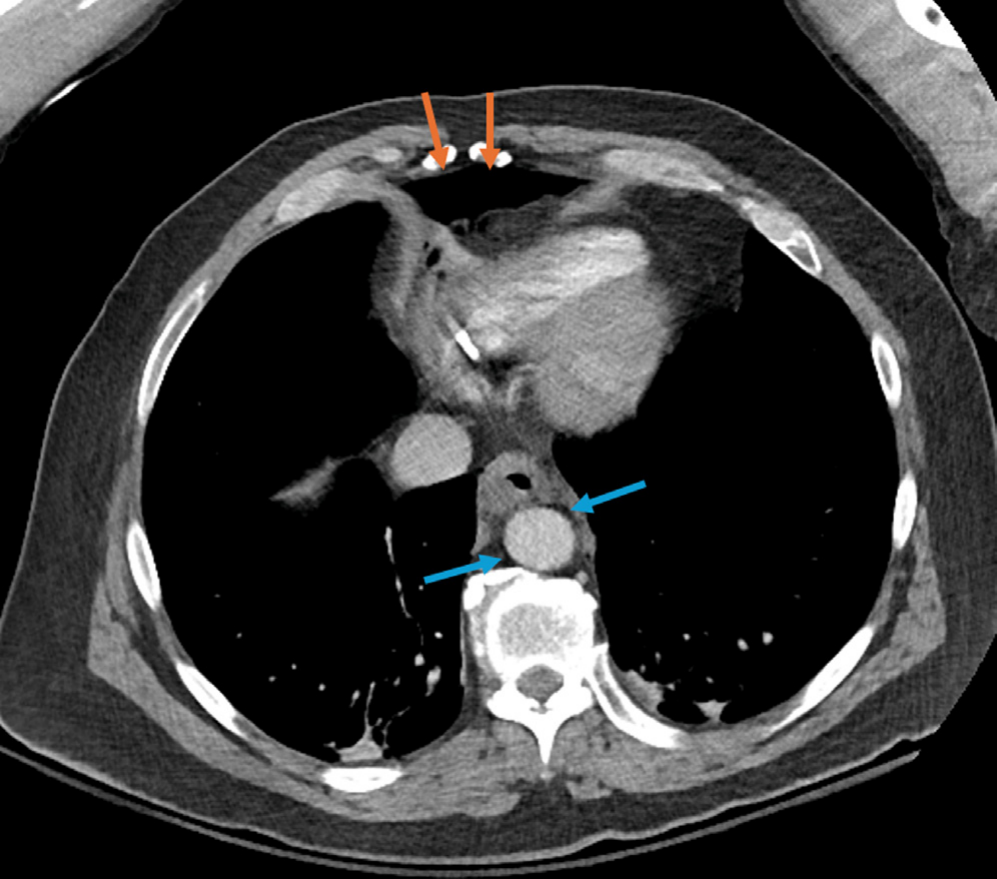
Computed tomography of the chest with intravenous contrast demonstrating pneumomediastinum (*orange arrows*) and retroperitoneal air (*blue arrows*).

**Figure 4:**
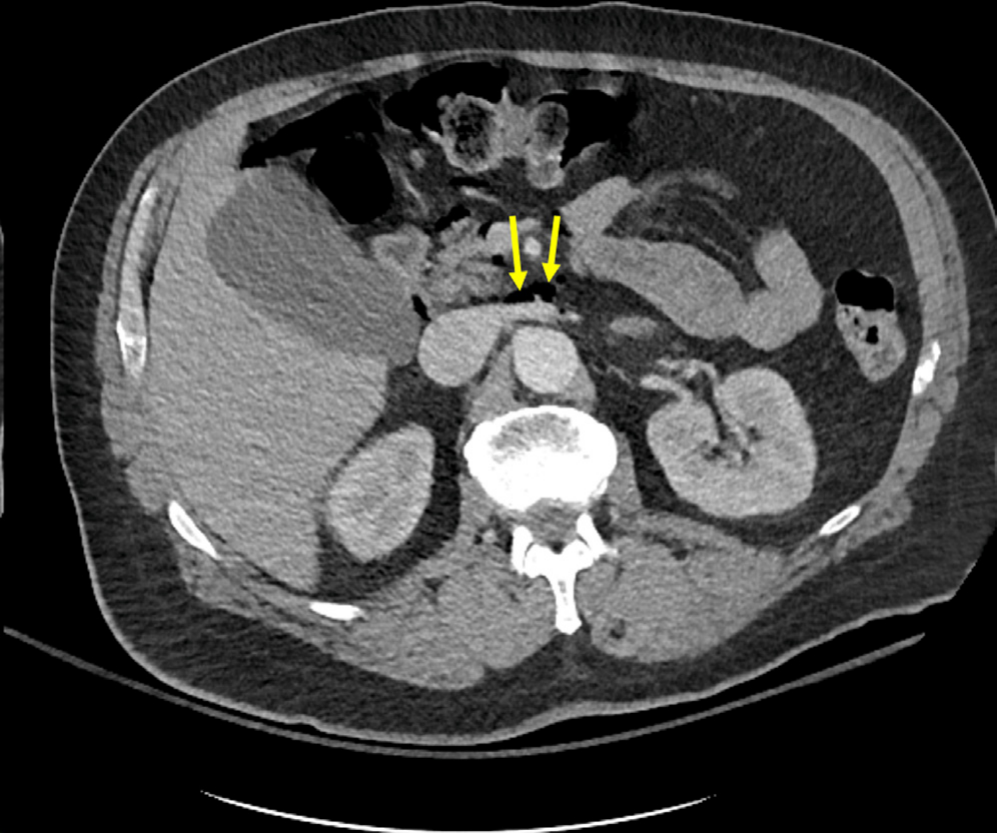
Computed tomography of the abdomen with intravenous contrast demonstrating a small volume of retroperitoneal air (*yellow arrows*).

Early AEs may be seen in one-quarter of patients undergoing POEM; however, serious AEs only occur in approximately 2.0% of cases.^1^ Notably, serious AEs are more common in patients with submucosal fibrosis, which has been associated with prior interventions before POEM, including botulinum toxin injection.^2^ Pain is common and may be observed in three-quarters of patients.^1^ An understanding of the expected radiographic findings post-POEM is important when evaluating a potential AE. Pneumomediastinum and pneumoperitoneum are the most common findings and are seen on cross-sectional imaging following POEM in most patients.^3^ Retroperitoneal free air is less common and may be seen in one-third of the patients.^3^ A small study examining routine esophagogram findings following POEM identified retroperitoneal free air in 40% of cases that resolved without intervention on follow-up imaging.^4^ The clinical utility of routine postprocedural imaging is of uncertain benefit.^5^ However, imaging should be pursued in patients with severe and unexplained symptoms with the understanding that significant radiographic findings are expected.

Our patient's symptoms improved with intravenous analgesics and nearly resolved by day 1 postprocedure. No esophageal leak was seen on the esophagogram obtained on day 1 postprocedure, and the patient was discharged. Our case highlights that retroperitoneal free air can be seen post-POEM, and these findings may not warrant further intervention in the absence of an esophageal leak identified on imaging, persistent fever despite conservative management, hemodynamic instability, or otherwise clinically indicated changes on evaluation.

## Patient consent

The patient in this article has given written informed consent to publication of their case details.

## Disclosure

The following author disclosed financial relationships: M. Bilal: Consultant for Boston Scientific, STERIS Endoscopy, Aspero Medical, and Cook Endoscopy. All other authors disclosed no financial relationships.
